# Contrasting effects of prolonged drought and nitrogen addition on growth and non-structural carbohydrate dynamics in coexisting *Pinus koraiensis* and *Fraxinus mandshurica* saplings

**DOI:** 10.48130/forres-0025-0002

**Published:** 2025-02-11

**Authors:** Xiaoyu Wang, Deliang Lu, Leonie Schönbeck, Yini Han, Shangbin Bai, Dapao Yu, Qingmin Han, Qing-Wei Wang

**Affiliations:** 1 Jiyang College, Zhejiang A&F University, Zhuji 311800, China; 2 Key Laboratory of Forest Ecology and Management, Institute of Applied Ecology, Chinese Academy of Sciences, Shenyang 110016, China; 3 Southern Swedish Forest Research Centre, Swedish University for Agricultural Sciences, Lomma 23422, Sweden; 4 Western Slope of Changbai Mountain National Field Research Observation Station of Forest Ecosystem, Baishan 134506, China; 5 Forestry and Forest Products Research Institute (FFPRI), Matsunosato, Tsukuba, Ibaraki 305-8687, Japan

**Keywords:** Broad-leaved-Korean pine forest, Carbon storage, Drought acclimation, Nitrogen deposition, Drought stress

## Abstract

Global change drivers, including drought and nitrogen (N) deposition, exert a wide-ranging influence on tree growth and fitness. However, our current understanding of their combined effects is still limited. Non-structural carbohydrate (NSC) storage is an important physiological trait for tree acclimation to drought. It acts as an important mobile carbon reserve to support tree function when carbon fixation or transport are reduced under drought. It is crucial to investigate how tree species with different NSC storage characteristics (e.g., storage level, partitioning) respond to drought events, and how N alters these patterns. We investigated the combined effects of drought (80% reduction in precipitation) and N addition (0, 30, and 120 kg/ha/year) on the growth and NSC storage of *Pinus koraiensis* and *Fraxinus mandshurica* (dominant species in the forests of Northeast China) saplings over two consecutive growing seasons. The results indicated that *P. koraiensis* exhibited high tolerance to drought, with growth unaffected by drought alone until the mid-growing season in the second year. However, N addition reversed its drought acclimation by impairing root development and exacerbating carbon shortage. In contrast, *F. mandshurica* was sensitive to drought, it had significantly reduced growth at harvest despite a large amount of NSC accumulation. The present study highlights the contrasting effects of N deposition on drought adaptation in coexisting conifer and temperate broadleaf species, the conifer showing a higher risk of carbon deficiency with increasing N deposition (i.e., a stronger reversal effect of N addition), whereas an earlier cessation of growth under drought defines a larger carbon safety margin for broadleaved species. These results have important implications for the development of adaptive forest management strategies such as to enhance the protection of conifers in the context of global change.

## Introduction

Climate change, including more frequent and extreme events, has caused widespread negative impacts on forest structure and function^[1]^. In recent decades, extreme drought events have led to an increasing number of forest decline and tree mortality events across different forest types and biogeographic regions, posing a serious threat to regional and global ecological security^[2−6]^. Atmospheric nitrogen (N) deposition as another major form of global change has been shown to influence tree drought adaptation by affecting various functional traits and physiological processes^[7,8]^. However, our current understanding of the interaction of drought and N deposition effects on tree growth and fitness is still limited compared to single-factor effects (drought or N)^[9−12]^.

Nitrogen is an essential component for tree growth and function, and acts as the most common limiting nutrient element in terrestrial ecosystems^[13]^. The growth (biomass allocation) and carbon balance of drought-stressed trees can be altered by increased N application or atmospheric N deposition^[14,15]^. Prolonged drought could lead to poor root N uptake capacity and show negative feedback on tree C balance, which could be alleviated by soil N supply, as was shown for example in *Pinus sylvestris*^[16]^. N supply could enhance antioxidant defense levels to increase superoxide dismutase (SOD) and peroxidase (POD), and improve N assimilation in drought-stressed Chinese fir seedlings^[17]^. The predisposition of trees to increased N before drought could also affect the root system development, make trees more susceptible to hydraulic constraints^[7,18]^, increase C allocation to growth and respiration at the expense of NSC storage, and decrease root NSC storage^[19]^. The negative effect of drought on the radial growth of *Fagus sylvatica* was amplified by N fertilization but was not shown in *Quercus petraea* and *Pseudotsuga menziesii*^[11]^. These studies show that N affects drought acclimation differently in different tree species, which may be related to the species' intrinsic carbon fixation efficiency and carbon storage characteristics (e.g., storage level, partitioning), which have not been well addressed in current research^[20]^.

Non-structural carbohydrates (NSC), which are primarily starch and mobile sugars, serve as the primary long-term carbon (C) reserves in trees. They play a crucial role in providing a buffer against stressors^[21−27]^. In addition to supporting structural growth and maintaining fundamental metabolic respiration^[28,29]^, trees utilize stored NSC to maintain osmoregulation and to repair adjacent xylem conduits, thereby ensuring the continued hydraulic function of trees under drought conditions^[30,31]^. Stable and sufficient NSC in the storage organs are also important carbon sources for growth recovery after disturbance^[32−34]^. When the NSC storage is too low or when remobilization is limited due to transport failure, trees will eventually decline in growth or even die due to restricted carbon supply^[1,4,35]^.

Different tree species exhibit divergent NSC storage characteristics in terms of storage level, storage allocation, and seasonal change patterns^[22,36,37]^. The NSC allocation patterns in trees are likely to influence the mobilizable and available NSC levels (as a buffer) of trees exposed to disturbance events such as drought and further affect tree growth and survival^[38]^. Conifers have been considered to have high NSC storage in needles ('expensive' foliage) but lower average NSC levels in the stem and belowground, and less fluctuation across tissues and organs than deciduous broadleaved trees^[39,40]^. They are also more vulnerable to drought-induced carbon deficiency, compared to broadleaved species^[1,41]^. In contrast, deciduous ring-porous species require large energy investment in new foliage, need to renew their main transport system each spring due to the cavitation of the previous year's early wood vessels^[42,43]^, and thus have a much higher seasonality in their NSC demand than trees with other functional types^[44]^. Therefore, cross-seasonal observations are needed to understand how NSC storage and partitioning strategies of different tree species affect the NSC remobilization under drought conditions (short- and long-term responses), and to explore how N deposition affect these processes, which will help to provide a deeper understanding of differences in tree drought acclimation.

The Northeast region of China belongs to the north temperate climate zone with a relatively cool climate. However, from 1961 to 2017, the average annual warming rate in this region reached 0.31 °C per decade, which is higher than the national average warming rate in the same period and also the global warming rate in the past 50 years. The significant warm-dry trend makes it a hotspot of forest drought^[45]^. In addition, the regional total N deposition level has reached a high level of 15−25 kg/ha/year, which is much higher than other regions in Northeast Asia^[46]^. The mixed broadleaved Korean pine forest is the dominant forest type in this region and plays an important role in maintaining regional ecological security and high economic value. The Changbai Mountain region is one of its core distribution areas, which distributes a large area of primary forest^[47]^. However, in both primary and degraded secondary Korean pine forests, *P. koraiensis* (the dominant pine species) exhibits barriers to natural regeneration, which puts it at a competitive disadvantage compared to other dominant species such as broadleaved *F. mandshurica* (the dominant broad-leaved ring-porous broadleaved species). However, it is not clear how climate change factors (mainly warm and dry climates and N deposition) affect the adaptation of different tree species, and how the adaptation of different tree species will change in the context of future climate change.

Using *P. koraiensis* and *F. mandshurica* saplings, we assessed how NSC allocation, N supply status, and growth response to drought interacted with three N application levels (simulating atmospheric N input level) over two consecutive growing seasons, focusing on whether treatment-induced growth slowdown and whether the response was caused by carbon supply limitation. We aimed to test the hypothesis that: (1) drought-stressed *P. koraiensis* would show higher NSC depletion than *F. mandshurica*, considering that pine species are generally reported to have lower NSC storage levels and are susceptible to NSC deficiency under severe drought stress. In contrast, *F. mandshurica* might experience seasonal carbon dificiency due to its high C demand for growth in spring; and (2) increased N availability under drought causes different growth and carbon storage responses in different tree species. *F. mandshurica* may benefit more than *P. koraiensis* because broadleaved species tend to have greater NSC storage in non-photosynthetic organs (especially roots), which ensures that its root growth and function are less affected by N addition-induced carbon allocation to aboveground tissues.

## Materials and methods

### Study area

This study was conducted in the experimental field of Changbaishan Xipo National Field Observation and Research Station for Forest Ecosystem, Lushuihe Forestry Bureau, Jilin Province, China (42°56' N, 127°77' E, 520 m above sea level). The study area has a temperate continental climate, the average annual air temperature ranges from −7 to 3 °C, with a frost-free period of nearly 100 d, and the average annual precipitation ranges from 700 to 1,400 mm (60%−70% falls in June and July), with a high inter-annual variability in total precipitation amount.

### Experimental design

In December 2016, *P. koraiensis* and *F. mandshurica* saplings (both two years old, local provenance) from the nearby Lushuihe Seed Garden of the Forestry Bureau were transported and prepared for this study (N = 90 for each species). *P. koraiensis* saplings were 32.69 ± 6.00 cm in height, 0.55 ± 0.12 cm in basal diameter; *F. mandshurica* were 71.07 ± 10.54 cm in height, 0.81 ± 0.11 cm in basal diameter. The saplings were potted in a mixture of sparse forest-grassland soil mixed with sand in a ratio of 2:1, pot size was ~ 5.2 L in volume which was set based on root depth and crown width of saplings with older age at the nursery seed garden. The physical and chemical properties of the soil are given in Supplementary Table S1. The saplings were transferred to a rainproof greenhouse covered with a sunlight board with 80% light transmittance, to eliminate natural rainfall inputs, while maintaining a light level close to natural levels. In early July 2017, saplings of each species were randomly assigned to a combination of drought and N addition treatments as follows.

A randomized block design was adopted in the experiment to manipulate both drought and N application treatment effects on *P. koraiensis* and *F. mandshurica* saplings. The drought treatment has two levels: the average annual rainfall record of the last ten years was taken as the control (CK, average annual 790 mm), and 20% of this amount was taken as the drought treatment (Drought); the intensity is set according to the 10% most severe droughts in the last 15 years record. The N addition treatment contains three levels: control (N0), a low N addition level at 30 kg/ha/year (N1) which is close to the current N deposition levels in the Changbai region^[46]^, and a high N deposition level of 120 kg/ha/year (N2). NH_4_NO_3_ solution was added to the soil once every month from July to September 2017 and from June to July 2018 along with water treatment. In total, this resulted in six different interaction levels between drought (two levels: CK, Drought) and N application (three levels: N0, N1, N2) treatments, N = 15 per treatment. Air and soil temperature and humidity were automatically recorded by soil moisture and temperature loggers (Hobo Data Logger, Onset Computer).

### Harvest work and sample collection

Harvesting occurred during the growing season, specifically in August (S1) and September (S2) of 2017; during the dormant season, January 2018 (S3); and in the following years' early and mid-growing season - June (S4) and August (S5) 2018. Note, the *F. mandshurica* shows bud from May and leaf senescence at the end of October. These sampling time points were chosen based on the following considerations. Both previous manipulative experimental studies^[1,48]^ and preliminary experiments have shown that drought-stressed tree saplings generally survive the first drought growing season, but begin to show severe dysfunction in the second year. It has also been observed in some studies that tree saplings start to under-utilize carbon in the winter of the current drought year^[33,48]^. Therefore, from the perspective of understanding long-term drought adaptation (survival and acclimation), we focused on whether these two studied species experienced carbon starvation during the subsequent dormant period, how they performed in the second year (exhibited growth slowdown), and whether this response was caused by carbon supply constraints.

At each sampling time, three saplings from each D × N treatment were fully harvested. The entire sapling was then carefully excavated and subsequently separated into leaf, shoot, and root parts. For pine, the unfallen dead needles were not included. All samples were heated in an oven at a temperature of 80 °C within 2 h of collection to minimize biological activity, and then oven-dried to a constant mass at 65 °C for 48 h. All samples were weighed to get the dry biomass data. After that, the oven-dried material was ground to a fine powder and stored at 4 °C for further chemical analysis.

### Chemical determinations

NSC was defined as the percentage (%) of mobile sugars (glucose, fructose, and sucrose) and starch, and was determined using the enzymatic hydrolysis method which is modified from the previous methods^[49,50]^, and described in detail in previous studies^[33,51]^. About 10−12 mg dried material was boiled in 2 mL distilled water for 30 min, then 500 mL of the extract (including dissolved sugars and starch) were incubated with a fungal-produced amyloglucosidase from *Aspergillus niger* (Sigma-Aldrich, USA) for 15 h at 49 °C to digest starch into glucose to determine total NSC. The amyloglucosidase was dissolved as 5 mg/mL 0.1 M Na-acetate-buffer solution. For soluble sugars determination, an aliquot of 200 mL was taken from the extract after centrifugation and treated with Invertase and Isomerase (Sigma-Aldrich, USA) to degrade sucrose and convert fructose into glucose. The total amount of glucose in each sample was determined photometrically at 340 nm in a 96-well microplate photometer (Thermo Fisher Scientific, Finland) after enzymatic conversion to gluconate-6-phosphate (hexokinase reaction). The concentration of starch was calculated as total NSC minus sugars. Pure starch and glucose-, fructose-, and sucrose- solutions (1 mg/mL) were used as standards. For each sample, two replicate measurements were conducted to ensure reliability in NSC quantification (standard deviation ≤ 0.8). Variability in enzymatic hydrolysis is taken into account when improving the method (iodine solution was performed to test starch residue), and the data detected in this study did not exceed the maximum detection limit. NSC concentrations are expressed on a percent dry matter basis.

The N content was determined with an Element analyzer (Element Analyzer, vario MACRO cube, Germany), with measurement accuracy of ≤ 0.1%. Above 5−6 mg of ground plant material was weighed into tin capsules that were combusted in an element analyzer for chemical analysis.

### Statistical analyses

A weighted mean concentration of NSC, sugars, and N in different organs was calculated for each individual using the following formula, to roughly assess the potential maximum available carbon and N level of the entire plant^[52]^:

$ \rm Weighted_{content}=\dfrac{\begin{array}{c}\rm (Leaf_{content} \times Leaf_{biomass} + Shoot_{content} \times\\ \rm Shoot_{biomass} + Root_{content} \times Root_{biomass})\end{array}}{(Leaf_{biomass} + Shoot_{biomass} + Root_{biomass})}$

For *F. mandshurica* during the dormant season, leaf biomass, and chemical data were excluded.

The drought-stressed *P. koraiensis* had lower needle biomass at the final harvesting time (S5) than former time (S4), thus, the needle loss rate was estimated as: Needle loss rate (%) = (Needle mass_s4 _− Needle mass_s5_)/Needle mass_s4_ × 100%. This rate was caculated separately for trees at three different N addition levels.

Three-way ANOVA was first conducted to test sampling time, drought (D), N addition (N), and their interaction effects (D × N) on NSC concentrations and N content in different organs (NSC results shown in Supplementary Table S2). Significant seasonal variations in total NSC, sugar, and starch in all tissues were observed in both species. Therefore, the effects of drought and N treatments on NSC were considered separately within each sampling season. Two-way ANOVA was used to test drought (D), N addition (N), and their interaction effects (D × N) on NSC concentrations, dry biomass, N contents in different organs, weighted mean NSC, and N at individual levels on each sampling time. Supplementary Table S3 gives the complete ANOVA results for N contents. Multiple comparison analyses (Tukey post-hoc test) were carried out to examine differences between each D and N treatment combination. Partial Eta squared measurement was used to evaluate the effect size (the amount of variance accounted) of each independent variables^[53]^. Correlation analysis was applied to explore how mean NSC concentration changes with N content at the individual level. All statistical tests were conducted at the 0.05 level of significance.

Factor analysis of mixed quantitative and qualitative data (FAMD) was a principal component method dedicated to exploring data with both continuous and categorical variables. FAMD can be roughly considered as a mix between Principal Component Analysis (PCA) for numerical variables, and Multiple Correspondence Analysis (MCA) for categorical variables^[54]^. The continuous variables are scaled to unit variance and the categorical variables are transformed into a disjunctive data table (crisp coding) and then scaled using the specific scaling of MCA. This ensures the balance of the influence of both continuous and categorical variables in the analysis, which means both variables are on an equal footing to determine the dimensions of variability. This allows the study of the similarities between individuals taking into account mixed variables and study of the relationships between all the variables^[55]^. In this study, FAMD was performed to compare biomass allocation, NSC, and N storage similarity between different drought and N treatments, with 'FactoMineR' and 'factoextra' packages in R. All the statistical tests and figures were done with R software (R Development Core Team, 2023, Vienna, Austria), under the RStudio environment.

## Results

### Growth response

**Figure 1:**
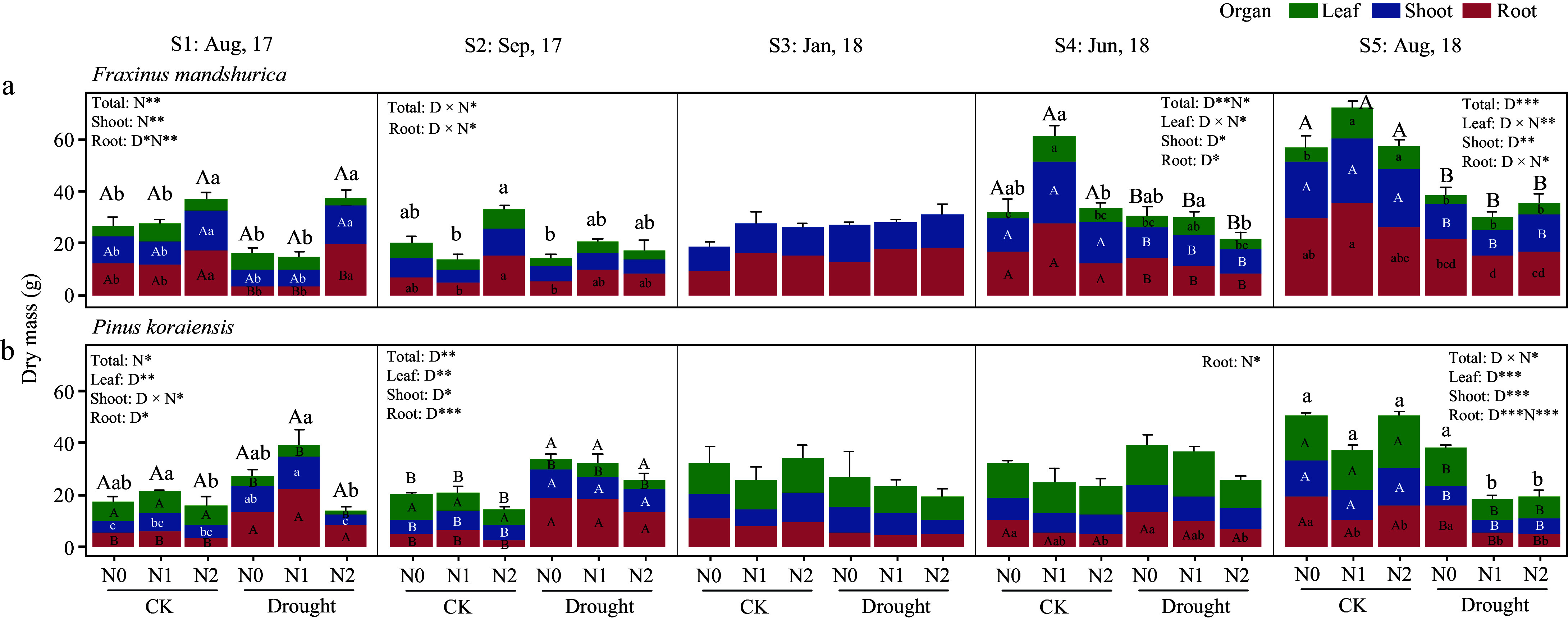
The growth response of *Fraxinus mandshurica* and *Pinus koraiensis* saplings under drought and combined nitrogen fertilization treatments. CK means the ambient precipitation and Drought means 20% of ambient amount. Bars and segments indicate mean ± se (n = 3 individuals) dry mass of each organ (colored). Different upper-case letters represent significant differences in means of biomass of total (on stacked bar) or each organ (inside bar) among drought treatments (D), while lower-case letters represent significant differences between nitrogen treatments (N), or between all six combinations in case of a D × N interaction exists, tested with Tukey post-hoc test. The ANOVA results for each organ and the total biomass were listed above each sub-figure. ***, **, and * indicates significant difference at *p* < 0.001, *p* < 0.01, and *p* < 0.05, respectively.

For *F. mandshurica*, one month after the start of the drought treatment (S1), the drought-induced root biomass reduction seemed to be alleviated by high N treatment (N2). This trend was observed in the following month as well (S2, for both N1 and N2 under drought) (Fig. 1, Table 1). In contrast, *P. koraiensis* showed reduced leaf biomass and increased root biomass in response to drought (also increased shoot growth in S2), which resulted in an overall increased individual biomass of *P. koraiensis*. During the dormant season (January 2018, S3), drought and N treatments did not lead to a significant growth decline for both species.

**Table 1:** Two-way ANOVA analysis of total, leaf, shoot and root dry biomass in *Fraxinus mandshurica* and *Pinus koraiensis* affected by drought and nitrogen addition treatments.

Species	Time	Factors	Total			Leaf			Shoot			Root	
			*F* value	*PEta*		*F* value	*PEta*		*F* value	*PEta*		*F* value	*PEta*
*F. mand*	S1: Aug, 2017	Drought (D)	4.482	0.272		0.005	0.000		2.746	0.186		5.437*	0.312
		Nitrogen (N)	8.815**	0.595		1.446	0.194		9.148**	0.604		12.093**	0.668
		D × N	1.397	0.189		0.606	0.211		0.369	0.058		3.245	0.351
	S2: Sep, 2017	Drought (D)	2.626	0.180		3.631	0.232		2.672	0.182		0.478	0.038
		Nitrogen (N)	3.230	0.350		0.768	0.113		1.359	0.185		4.304*	0.418
		D × N	4.856*	0.447		1.554	0.206		3.364	0.359		4.039*	0.402
	S3: Jan, 2018	Drought (D)	0.638	0.050					3.884	0.245		0.606	0.048
		Nitrogen (N)	0.268	0.043					0.188	0.030		1.413	0.191
		D × N	0.083	0.014					2.220	0.270		0.032	0.005
	S4: Jun, 2018	Drought (D)	9.920**	0.453		1.303	0.098		8.693*	0.420		8.108*	0.403
		Nitrogen (N)	5.459*	0.476		29.768***	0.832		2.696	0.310		3.732	0.383
		D × N	3.371	0.360		5.865*	0.494		2.283	0.276		2.502	0.294
	S5: Aug, 2018	Drought (D)	41.649***	0.776		66.196***	0.847		17.486**	0.593		48.484***	0.802
		Nitrogen (N)	0.433	0.067		17.308***	0.743		0.054	0.009		2.259	0.273
		D × N	3.057	0.338		7.592**	0.559		0.678	0.102		4.982*	0.454
*P. Kora*	S1: Aug, 2017	Drought (D)	3.669	0.234		16.656**	0.581		15.063**	0.557		7.896*	0.397
		Nitrogen (N)	3.949*	0.397		1.008	0.144		9.685**	0.617		2.008	0.251
		D × N	1.702	0.221		0.356	0.056		5.614*	0.483		1.018	0.145
	S2: Sep, 2017	Drought (D)	15.113**	0.557		10.300**	0.462		7.209*	0.375		53.981***	0.819
		Nitrogen (N)	2.095	0.259		2.205	0.269		0.028	0.005		2.998	0.333
		D × N	0.079	0.013		1.086	0.153		0.875	0.127		0.265	0.042
	S3: Jan, 2018	Drought (D)	0.822	0.064		0.320	0.026		0.237	0.019		2.283	0.160
		Nitrogen (N)	0.126	0.021		0.029	0.005		0.249	0.040		0.182	0.029
		D × N	0.210	0.034		0.137	0.022		1.106	0.156		0.061	0.010
	S4: Jun, 2018	Drought (D)	2.492	0.172		1.624	0.119		0.374	0.058		4.425	0.269
		Nitrogen (N)	2.070	0.256		1.411	0.190		0.505	0.078		5.206*	0.465
		D × N	0.401	0.063		0.696	0.104		0.145	0.024		0.195	0.031
	S5: Aug, 2018	Drought (D)	74.837***	0.862		27.466***	0.696		34.786***	0.744		31.475***	0.724
		Nitrogen (N)	15.603***	0.722		3.147	0.344		0.208	0.230		23.644***	0.798
		D × N	5.328*	0.470		3.758	0.385		0.683	0.062		3.546	0.371
*F* value of ANOVA is given, ***, **, and * indicates significant difference between different treatment levels at *p* < 0.001, *p* < 0.01, and *p* < 0.05, respectively (*F* value in bold if *p* < 0.05). *F. mand*: *Fraxinus mandshurica*, *P. kora*: *Pinus koraiensis*. *PEta*: Partial Eta squared which used to measure the effect size of different variables in ANOVA models.													

During the second growing season, *F. mandshurica* showed high sensitivity to drought, while *P. koraiensis* was affected by both factors. At the final harvest time (S5), drought decreased total biomass of *F. mandshurica* by 44.13%. While shoot biomass significantly decreased due to drought, leaves and roots were affected by an interaction of drought and N addition, but results show ambiguous patterns that do neither point to mitigation, nor exacerbation of drought impacts on its growth. As for *P. koraiensis*, its total biomass was significantly affected by the drought-N interaction effect. Drought alone (N0 under drought) did not lead to growth decline, but N addition under drought (for both N1 and N2 under drought) decreased the total biomass by 62.9% (compared to N0 under CK). Both leaf and shoot growth were significantly decreased by drought, whereas root growth was reduced by both drought and N treatments (*p* < 0.001) (Fig. 1, also see Supplementary Fig. S1 for relative biomass allocation). The needle loss rate of drought-stressed trees (harvest S5 compared to S4) reached 3.5%, 54.1%, and 21.8% at N0, N1, and N2 nitrogen levels respectively.

### NSC allocation response

**Figure 2:**
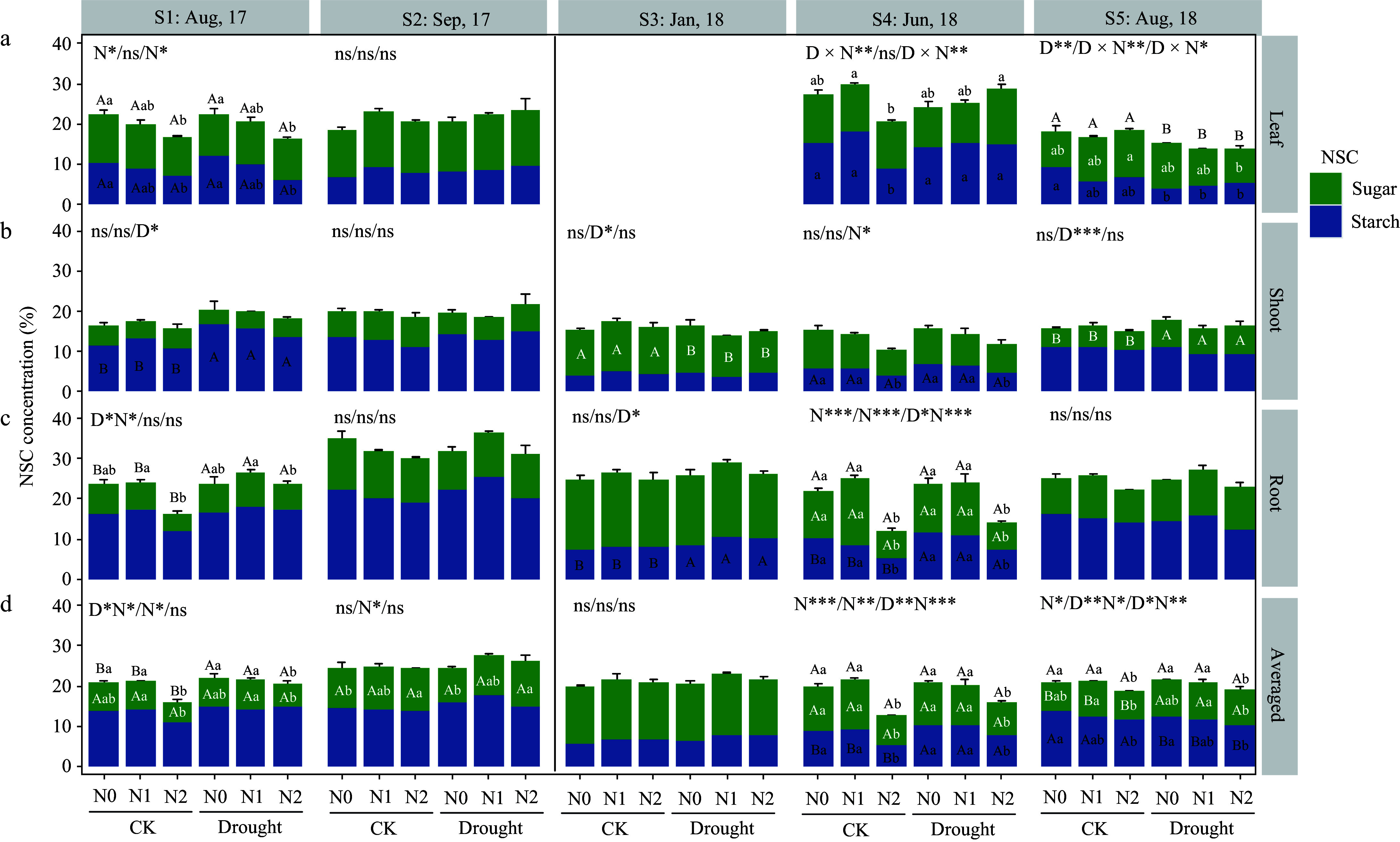
Non-structural carbohydrate (NSC) response in different organs of *Fraxinus mandshurica* saplings at different sampling times. Bars and segments indicate mean ± se (n = 3 individuals) NSC concentration, different upper-case letters represent significant differences in means (on stacked bar for total NSC, or inside bar for sugar or starch) among drought treatments (D), while lower-case letters represent significant differences between nitrogen treatments (N), or between all six combinations in case of a D × N interaction exists, tested with Tukey post-hoc test. The ANOVA results for total NSC (sugar + starch), sugar and starch (delimited with slash symbol) were listed above each sub-figure. ***, **, and * indicates significant difference at *p* < 0.001, *p* < 0.01, and *p* < 0.05, respectively. ns means non-significant.

For *F. mandshurica*, heavy N addition (N2) caused total NSC reductions in leaves (S1 time), and roots (S1 and S4 times) in the summer seasons (Fig. 2). While in the autumn and dormancy season, NSC levels were kept stable. By the mid-growing season (S5), its leaf NSC was significantly reduced by drought, and total NSC in roots was kept at consistent levels among treatments, but soluble sugars in shoots were significantly higher under drought treatment.

**Figure 3:**
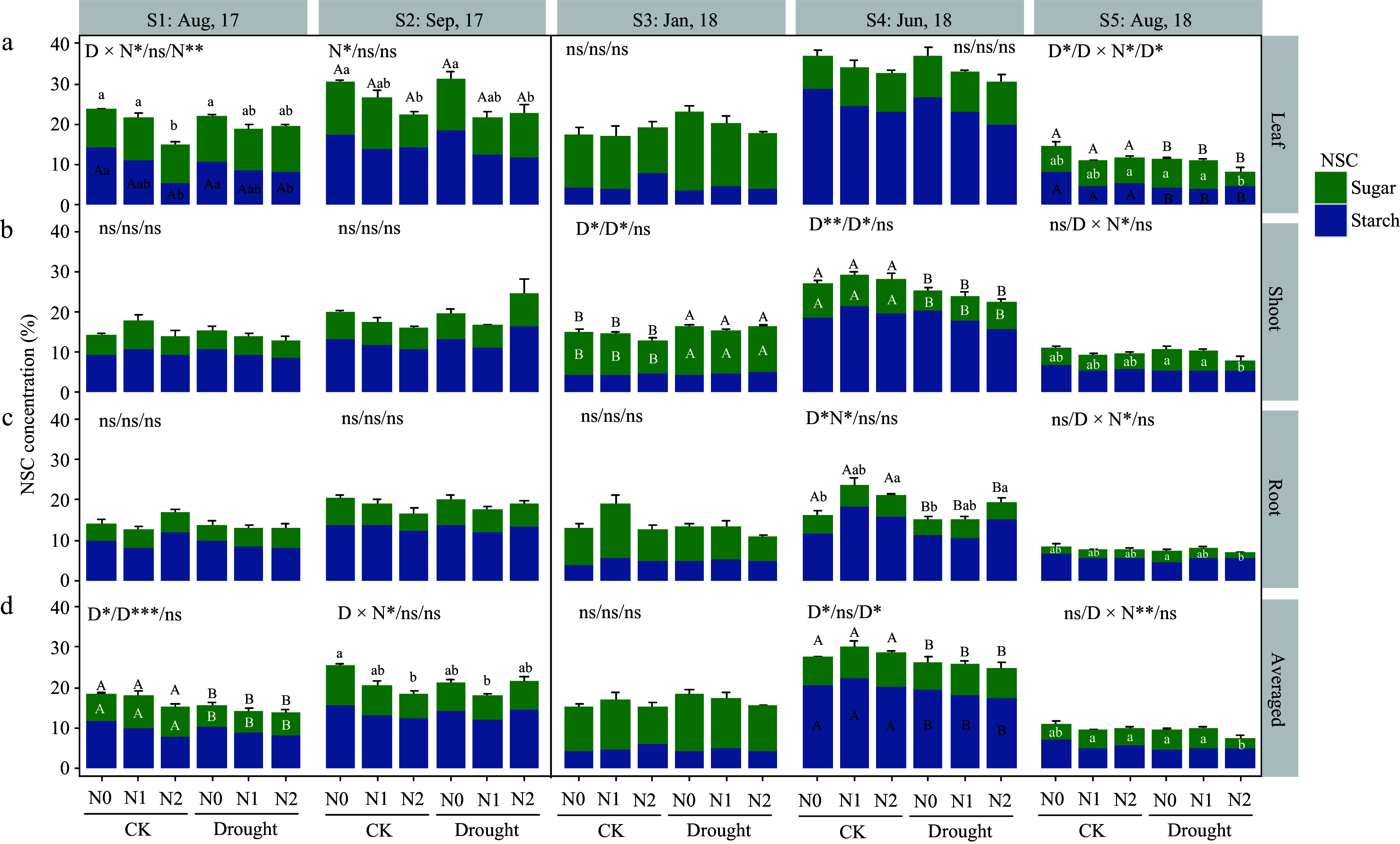
Non-structural carbohydrate (NSC) response in organs of *Pinus koraiensis* saplings at different sampling times. Bars and segments indicate mean ± se (n = 3 individuals) NSC concentration, different upper-case letters represent significant differences in means (on stacked bar for total NSC, or inside bar for sugar or starch) among drought treatments (D), while lower-case letters represent significant differences between nitrogen treatments (N), or between all six combinations in case of a D × N interaction exists, tested with Tukey post-hoc test. The ANOVA results for total NSC (sugar + starch), sugar and starch (delimited with slash symbol) were listed above each sub-figure. ***, **, and * indicates significant difference at *p* < 0.001, *p* < 0.01, and *p* < 0.05, respectively. ns means non-significant.

As in *P. koraiensis* in the first growing season (S1 and S2), only leaf NSC was reduced by high N treatment (N2). In the dormant season (S3), shoot NSC was significantly increased by drought (due to shoot sugar changes) (Fig. 3). At S4, shoot NSC (sugar) was significantly lower under drought, and root NSC was affected by N and drought interactions, N2 had a tendency to increase the total NSC. At harvest time (S5), drought caused a significantly decreased total NSC (because of starch) in leaves, besides, heavy N induced a decrease in shoot and root sugar accumulation in drought-stressed individuals.

From June to August (S4−S5), the weighted mean NSC of *P. koraiensis* decreased significantly in all the treatments, while that of *F. mandshurica* increased or was maintained (Figs 2d & 3d).

### The NSC, N storage, and growth relations in different species

**Figure 4:**
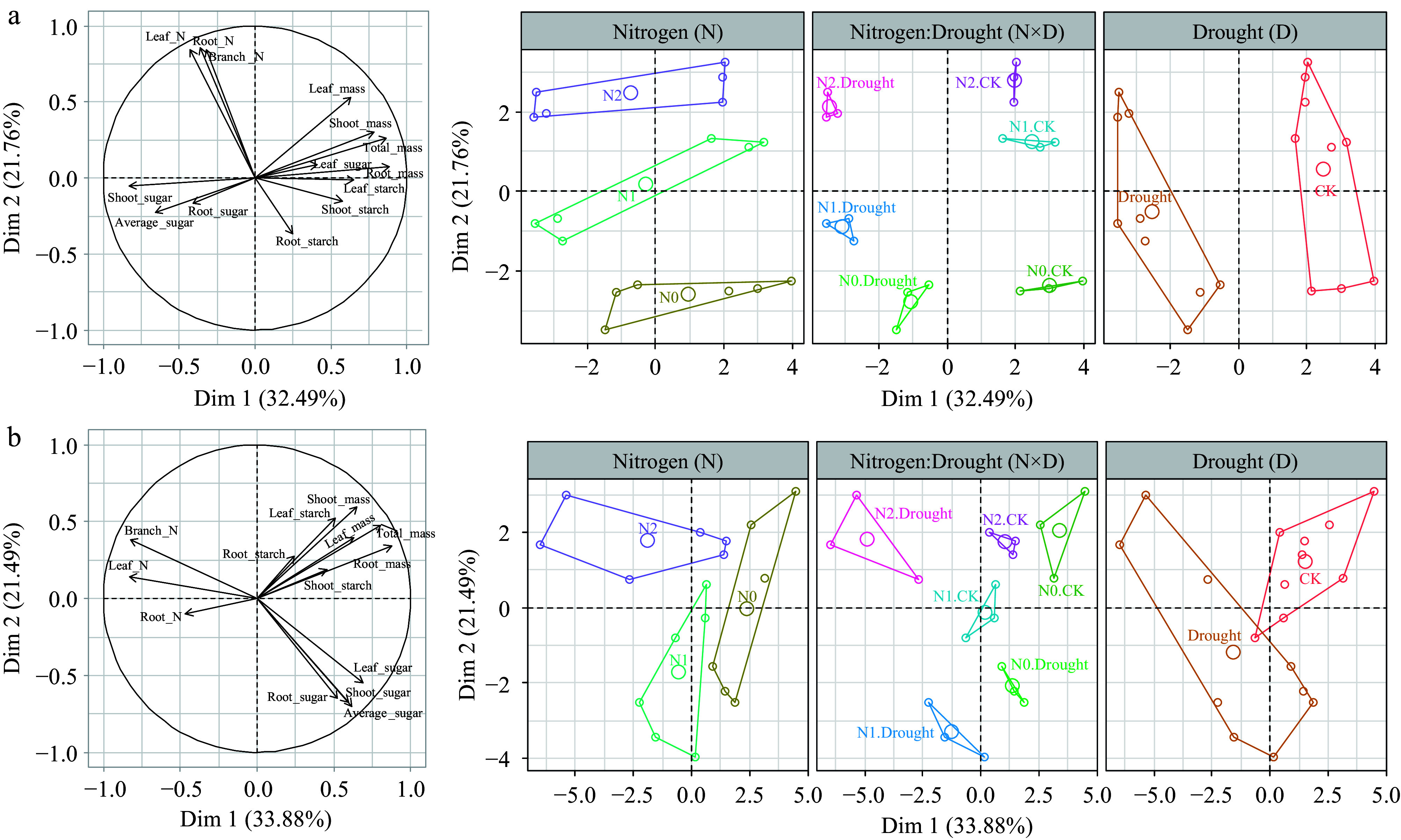
Factor analysis of mixed (FAMD) non-structural carbohydrate (NSC), nitrogen (N) content in relation to growth in *Fraxinus mandshurica* and *Pinus koraiensis* at the final harvest time.

The FMAD results show NSC, N storage, growth patterns, and their relationship with drought and N treatments in different species at final harvest time S5 (Fig. 4). For *F. mandshurica*, the first dimension was mainly explained by drought (32.49%), and was associated with a trend of sugar accumulation and growth reduction in drought-stressed saplings. Dimension 2 explained 21.76% and was mainly characterized by N treatments, associated with N accumulation in roots, leaves and shoots. For *P. koraiensis*, interactive effects of drought and N were found, within drought stressed individuals, N content was negatively correlated with sugar accumulation. *P. koraiensis* is significantly affected by drought-N interactions, which is not seen in *F. mandshurica*.

**Figure 5:**
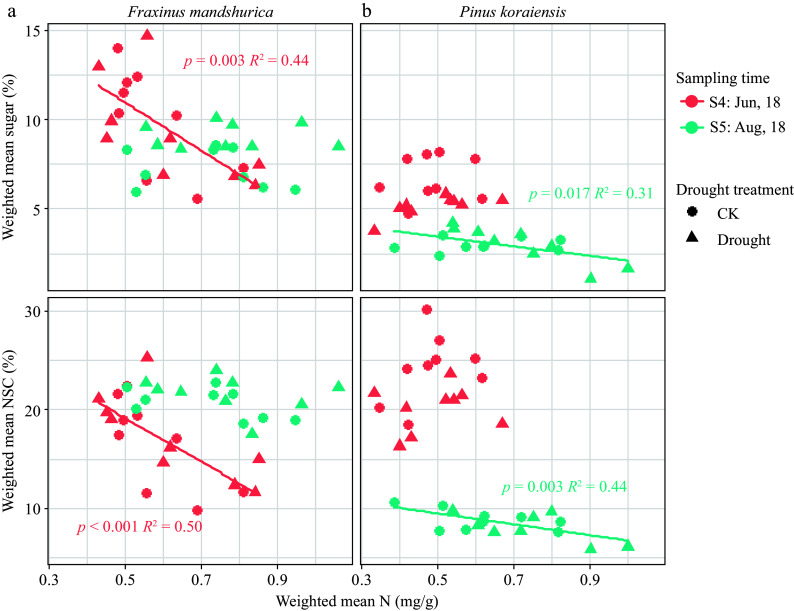
Weighted mean total NSC and sugar concentration scaled with N content for different species at different sampling times. Parameters of fitted linear model (with *p* < 0.05) are given on each sub-figure.

Consistent with this trend, the N content was largely increased by N addition and changed to a lesser extent by season, with drought only affecting leaf N content of two species (decreasing trend) (Tables 2 & 3). Supplementary Table S4 shows the detailed N content in woody tissues. For *F. mandshurica* in June 2018 (S4) and *P. koraiensis* in August 2018 (S5), sugar and NSC were all significantly negatively correlated with N content (Fig. 5).

**Table 2:** Three-way ANOVA analysis of sampling time, drought, and nitrogen (N) addition treatments effects on N content in different organs.

Measurements	Factors	*Fraxinus mandshurica*			*Pinus koraiensis*	
		*F* value	*PEta*		*F* value	*PEta*
Leaf N	Time (T)	5.261**	0.200		8.666***	0.302
	Drought (D)	8.530**	0.119		4.175*	0.050
	Nitrogen (N)	54.789***	0.635		21.438***	0.349
	D × N	0.074	0.002		1.171	0.028
Shoot N	Time (T)	14.977***	0.428		5.759***	0.224
	Drought (D)	2.398	0.029		3.089	0.027
	Nitrogen (N)	17.753***	0.307		17.265***	0.301
	D × N	0.700	0.017		0.484	0.012
Root N	Time (T)	10.773***	0.350		47.128***	0.702
	Drought (D)	2.513	0.030		2.118	0.026
	Nitrogen (N)	29.981***	0.428		16.025***	0.286
	D × N	0.495	0.012		1.788	0.043
Weighted N	Time (T)	7.175***	0.255		10.604***	0.346
	Drought (D)	0.012	0.000		0.410	0.005
	Nitrogen (N)	22.405***	0.416		33.934***	0.459
	D × N	0.977	0.030		0.011	0.000
*F* value of results is given, ***, **, and * indicates significant difference between different treatment levels at *p* < 0.001, *p* < 0.01, and *p* < 0.05, respectively (*F* value in bold if *p* < 0.05). *PEta*: Partial Eta squared which used to measure the effect size of different variables in ANOVA models.						

**Table 3:** Leaf and the whole plant weighed (averaged) nitrogen content (Mean and SD, N mg/g).

Sampling time	Drought treatment	Nitrogen treatment	*Fraxinus mandshurica*			*Pinus koraiensis*	
			Leaf N	Weighted N		Leaf N	Weighted N
S1:Aug, 2017	CK	N0	0.941 (0.195)c	0.206 (0.041)b		0.855 (0.059)a	0.603 (0.04)Ab
		N1	1.485 (0.158)b	0.578 (0.171)ab		0.706 (0.415)a	0.517 (0.13)Ab
		N2	2.054 (0.083)a	0.692 (0.191)a		1.253 (0.294)a	0.847 (0.152)Aa
	Drought	N0	1.1 (0.14)c	0.555 (0.025)ab		0.828 (0.279)a	0.462 (0.094)Bb
		N1	1.023 (0.034)c	0.441 (0.203)ab		1.181 (0.061)a	0.513 (0.023)Bb
		N2	1.472 (0.083)b	0.41 (0.265)ab		0.705 (0.152)a	0.629 (0.034)Ba
S2:Sep, 2017	CK	N0	0.931 (0.126)Ac	0.498 (0.156)Ab		0.724 (0.03)Ab	0.591 (0.014)
		N1	1.403 (0.164)Ab	0.889 (0.22)Ab		1.007 (0.189)Aab	0.627 (0.179)
		N2	1.853 (0.135)Aa	0.994 (0.232)Aa		1.184 (0.198)Aa	0.813 (0.136)
	Drought	N0	0.699 (0.187)Ac	0.246 (0.043)Ab		0.964 (0.435)Ab	0.531 (0.216)
		N1	1.072 (0.071)Ab	0.539 (0.138)Ab		1.095 (0.252)Aab	0.714 (0.141)
		N2	1.852 (0.324)Aa	1.299 (0.442)Aa		1.319 (0.159)Aa	0.733 (0.137)
S3:Jan, 2018	CK	N0	*No data	0.307 (0.014)Ab		0.613 (0.065)Ab	0.435 (0.06)Bc
		N1		0.584 (0.217)Aab		1.159 (0.298)Aa	0.765 (0.17)Bb
		N2		1.176 (0.208)Aa		1.309 (0.179)Aa	0.781 (0.036)Ba
	Drought	N0		0.283 (0.062)Bb		0.968 (0.096)Ab	0.606 (0.019)Ac
		N1		0.444 (0.223)Bab		1.168 (0.188)Aa	0.707 (0.053)Ab
		N2		0.53 (0.42)Ba		1.488 (0.169)Aa	0.98 (0.105)Aa
S4:Jun, 2018	CK	N0	1.474 (0.396)Ab	0.54 (0.081)bc		0.627 (0.025)Ac	0.397 (0.042)Ab
		N1	1.118 (0.059)Ab	0.502 (0.026)c		0.696 (0.005)Ab	0.48 (0.014)Aa
		N2	1.701 (0.2)Aa	0.685 (0.128)ab		0.842 (0.02)Aa	0.573 (0.06)Aa
	Drought	N0	0.961 (0.128)Bb	0.45 (0.018)c		0.626 (0.068)Ac	0.395 (0.052)Ab
		N1	1.069 (0.054)Bb	0.593 (0.031)bc		0.756 (0.044)Ab	0.497 (0.085)Aa
		N2	1.352 (0.074)Ba	0.827 (0.034)a		0.862 (0.106)Aa	0.583 (0.075)Aa
S5:Aug, 2018	CK	N0	0.618 (0.122)Ac	0.528 (0.024)Ac		0.661 (0.042)Ab	0.468 (0.072)Ab
		N1	1.035 (0.131)Ab	0.75 (0.028)Ab		0.963 (0.206)Aab	0.672 (0.133)Aab
		N2	1.324 (0.131)Aa	0.872 (0.068)Aa		1.039 (0.202)Aa	0.72 (0.097)Aa
	Drought	N0	0.663 (0.096)Ac	0.596 (0.047)Ac		0.826 (0.028)Ab	0.6 (0.053)Ab
		N1	1.169 (0.105)Ab	0.762 (0.021)Ab		0.979 (0.205)Aab	0.686 (0.133)Aab
		N2	1.516 (0.244)Aa	0.953 (0.114)Aa		1.229 (0.098)Aa	0.884 (0.125)Aa
The upper-case letters represent significant differences in means among drought treatments, while lower-case letters represent significant differences between N treatments, or between all six combinations in case of a D × N interaction exists, tested with Tukey post-hoc test.							

## Discussion

The present results showed that *P. koraiensis* exhibits high drought tolerane with growth unaffected by drought treatments alone. N fertilization did not alleviate but exacerbated the negative effects of drought on its growth by increasing the carbon shortage (shoot and root sugar mostly affected) and impaired root development. In contrast, *F. mandshurica* shows an earlier cessation of growth under drought at which time a large amount of NSC was accumulated in the tissues. In the early growing season in the second year (June, 2018), high N fertilization resulted in lower NSC storage for both well-watered and droughted trees, but only drought-stressed *F. mandshurica* showed a growth decrease trend, suggesting that water limitation was more of a driving factor than carbon shortage. Thus, the present results support the hypothesis that *P. koraiensis* would experience higher NSC depletion than *F. mandshurica*, but not the hypothesis that *F. mandshurica* would experience more carbon shortage in spring (Hypothesis 1). Hypothesis 2 was accepted, given the greater attenuation of N to drought acclimation of *P. koraiensis*.

At the final harvest time after two growing seasons of drought, both species maintained similar total NSC levels in shoots and roots across treatments, despite different growth responses, but there is a significant difference in the actual available NSC allocated to growth. Both species had large biomass accumulation from June to August 2018 but showed different NSC change patterns. From the change pattern of averaged NSC at the whole-plant level, it can be inferred that the growth of *F. mandshurica* did not cause a decrease in NSC reserves (its averaged NSC remained stable, see Fig. 2d), while *P. koraiensis* showed a significant downward trend (averaged NSC decreased, Fig. 3d). Thus, drought-stressed *F. mandshurica* showed a passively accumulated or maintained NSC when growth declined, the overall C supply being sufficient. Whereas the *P. koraiensis* showed a relative C shortage, C allocation to growth was a strong carbon sink considering that the averaged NSC storage level decreased meaning a net reduction in the individual level NSC storage (C consumption higher than fixation). Plant tissues commonly adaptively maintain higher levels of sugar concentrations than the control under drought stress^[52,56,57]^. It seems however that *P. koraiensis* did not retain enough NSC in storage to cope under prolonged drought, with slowdown growth as an expense. Thus, stable NSC levels across treatments may be the result of a trade-off between storage and growth.

Compared to the total NSC, soluble sugar levels better described the actual sugar use for drought acclimation, considering that low molecular weight sugars are commonly accumulated in the tissues to aid osmotic adjustment and to repair xylem embolisms for drought-stressed plants^[31,58]^. In the present study, sugars in woody organs (shoots and roots) of *F. mandshurica* were indeed higher under drought stress, and the starch and total NSC concentrations in the tissues were also abundant (Fig. 2). Thus, the growth decline of *F. mandshurica* as a result of NSC or sugar shortage can be ruled out, as it is likely directly linked to water limitation (active slow down of growth or passive growth decrease due to water deficit). The drought-stressed *P. koraiensis* individuals (without N addition) also accumulated sugars in both shoots and roots, but in interaction with N addition (N2 level), sugar levels significantly decreased, while starch levels were unaffected (Fig. 3).

For *P. koraiensis*, it is unexpected that shoot and root starch storage was unaffected in drought-stressed individuals under the N addition treatment when root sugars were depleted. In contrast to this result, low root starch was found in many cases of weakened or dead deciduous and coniferous species^[19,59,60]^. This may be due to the inability to efficiently activate starch degradation under long-lasting water deficit and osmotic stress^[61,62]^, or the inhibition of starch conversion or sugar mobilization. We do not have data on this phenomenon in our current study, and this should be further clarified in future studies. The present results are in accordance with previous studies, that report that reduction in NSC at tree mortality is more prevalent for gymnosperms than for angiosperms. This occurred in over 90% of cases in temperate Pinaceae species, particularly in the roots^[1]^.

In the present study, the overall N supply in different organs was sufficient for both species studied (concentration did not decrease affected by drought). However, it was observed that N was significantly negatively correlated with sugar accumulation in the storage organs of *P. koraiensis* in the middle of the growing season (Fig. 5). This is consistent with some previous studies reporting that N addition tends to increase aboveground C investment and respiration, reduce NSC storage, especially in roots, and increase fine and coarse root mortality^[63]^, which will further increase evaporative demand and exacerbate water limitation^[12,19]^. N addition also reduces the tissue desiccation tolerance, increases transpiration, and stomatal sensitivity to close stomata at higher water potentials^[64]^, and lastly enhances susceptibility to drought-induced hydraulic failure^[65]^. In addition, N uptake requires more sugar consumption in the roots (through active absorption), which further impedes osmoregulation and may exacerbate the effects of drought^[66,67]^.

In this study, the growth slowdown of *P. koraiensis* caused by N addition under drought stress coincided with sugar deficiency. Thus, increased root biomass as an acclimation response to drought stress of *P. koraiensis* in the first year (short-term response) could not be maintained in the second year (long-term response, for interacting drought and N levels). As previous studies discussed, the role of nutrient availability before, during, and after drought varies considerably^[7]^. In this study, drought, and N treatments were applied simultaneously (representing the during-drought N effect). However, in actual forests, trees may exhibit changes due to high N deposition long before a drought event occurs, which will in turn, affect drought vulnerability. This needs to be considered in future studies.

Early spring growth of *F. mandshurica* was positively correlated with average wood tissue sugar concentration, especially in well-watered individuals (Supplementary Fig. S2), confirming that although there was no evidence of seasonal carbon deficiency due to drought, higher sugar allocation had a positive effect on growth resumption under well-watered conditions. This was also confirmed in mature *Quercus pyrenaica* that sapwood sugar concentrations are largely involved in growth resumption and xylem production in spring^[44]^. Noteworthy, N dramatically decreased NSC storage of *F. mandshurica* in the woody tissues (Fig. 5), but only interacting drought and high N fertilization treated individuals showed growth reduction, possibly due to moisture limitation being further amplified. But because of high C-fixation efficiency, the decreased NSC was fast replenished in the subsequent growth stage, thus no severe sugar or total NSC shortage occurred for *F. mandshurica*.

The higher drought sensitivity of *F. mandshurica* than *P. koraiensis* (for higher growth down-regulation by drought) could be explained by the hydraulic safety vs efficiency trade-off, where angiosperm species, especially ring-porous species with larger xylem conduits diameter and longer conduit length, have higher water-transport capacities and water use efficiencies to support faster growth rates, in turn however also leading to higher embolism vulnerability^[68−71]^. The results of tree species comparisons are also in line with a study on *Eucalyptus globulus* Labill. that an earlier cessation of growth under drought defines a wider 'carbon safety margin', compared to *Pinus radiata* showing sustained growth when NSC supply from photosynthesis decreased^[72]^. Trees as long-lived organisms will encounter periodicity and non-periodicity stress throughout their lifetimes^[73,74]^, and they have to allocate certain NSC storage to ensure survival, to be used as osmoregulation and embolism repair functions at the expense of growth^[75]^. However, the conifer species seems commonly reported to exhibit a lagging growth response (growth slowdown) to drought stress which is an important cause of NSC shortage in drought-weakened or dead conifer trees^[72,76,77]^. Understanding differences in the response of different functional groups of trees (angiosperms and gymnosperms, deciduous trees, and evergreens) to drought and N deposition will help to better predict changes in forests characterized by different tree species compositions in future climates^[73,74]^.

The findings of this study have to be seen in light of some limitations. Due to the large number of treatment combinations and observations were conducted across seasons, the number of replications assigned to each treatment type was relatively small, which may have weakened the statistical power to some extent. However, this study was based on a sufficiently large sample of a total of 180 saplings (90 of each species), and some key results, such as the NSC response of seedlings in the second drought year, showed a high degree of consistency across replicates. In addition, the hydraulic parameters will greatly improve our understanding of the complexity of drought responses in different tree species, which we will investigate more comprehensively in our future work.

The results of the present study are not 'completely new' findings, as the studied red pine ultimately showed carbon deficiency under sustained drought which is consistent with previous results^[1]^ (synthesis study), and the exacerbation of the carbon deficiency by N addition has been reported in some previous studies (mitigation drought stress for other cases). However, what is intriguing in the case of this study is that in the absence of interacted N additions, *P. koraiensis* has a relatively high drought tolerance, N becames a key component in reversing its drought adaptation and triggering carbon shortage (sugar deficiency). This is likely related to the growth regulatory mechanisms and biological traits of the studied species. Compared to *F. mandshurica* (earlier cessation of growth, and high carbon stocks), *P. koraiensis* had a less pronounced down-regulation of growth and its carbon storage was significantly reduced in the later stages of growth. This is likely related to long-lived and expensive foliage and low carbon fixation efficiency characteristics of this species.

## Conclusions

The present study argues that high nitrogen addition poses an additional risk of carbon starvation for *P. koraiensis*, also reversing its drought-adapted traits of high root biomass allocation. We cannot exclude the effect of lack of turgor pressure due to drought as a key factor in the growth decline. Drought superimposed on nitrogen fertilization also resulted in a substantial reduction of sugar in the storage organs of *F. mandshurica*, significantly aggravated water limitation, and slowed growth in the early growing season. When the drought persisted until the mid-growing season, total NSC, and sugar stores were replenished and growth was still largely determined by water availability. Thus, water sensitivity probably played a dominant role in the down-regulation of growth rates of *F. mandshurica* under drought conditions. Our results thus suggest that *P. koraiensis* saplings are at higher risk of drought adaptation being weakened by nitrogen deposition (i.e., a stronger N addition reversal effect), ultimately triggering a carbon deficit in this species and causing growth decline, while an earlier growth cessation under drought defines a larger carbon safety margin for *F. mandshurica*.

## SUPPLEMENTARY DATA

Supplementary data to this article can be found online.

## Data Availability

The datasets generated during and/or analyzed during the current study are available from the corresponding author on reasonable request.
