# Supplementary material for: Contrasting effects of prolonged drought and nitrogen addition on growth and non-structural carbohydrate dynamics in coexisting Pinus koraiensis and Fraxinus mandshurica saplings
Source: For Res (Fayettev). 2025 Feb 11;5:e003. doi: 10.48130/forres-0025-0002 (PMC11870304; doi:10.48130/forres-0025-0002)
Supplement: Supplementary file 1 — Supplementary data to this article can be found online. [file forres-0025-0002-S1.zip › 10.48130_forres-0025-0002-Suppl-FigureS2.pdf]

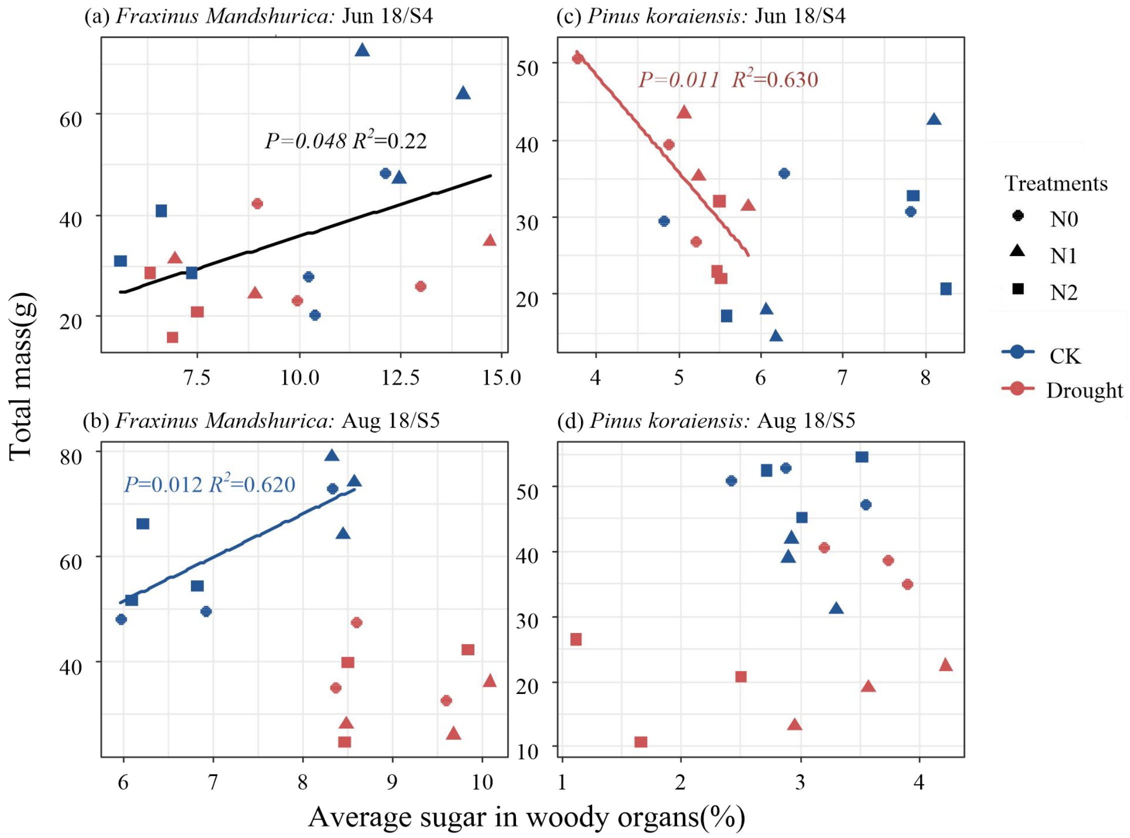

Fig. S2 The total biomass in relation to averaged sugar levels in the storage organs of *Fraxinus mandshurica* and *Pinus koraiensis*. In the sub-figure a, the fitted line in black color represents pooled data for CK and Drought individuals.
