# Supplementary material for: Contrasting effects of prolonged drought and nitrogen addition on growth and non-structural carbohydrate dynamics in coexisting Pinus koraiensis and Fraxinus mandshurica saplings
Source: For Res (Fayettev). 2025 Feb 11;5:e003. doi: 10.48130/forres-0025-0002 (PMC11870304; doi:10.48130/forres-0025-0002)
Supplement: Supplementary file 1 — Supplementary data to this article can be found online. [file forres-0025-0002-S1.zip › 10.48130_forres-0025-0002-Suppl-TableS1.pdf]

Table S1. Soil physical and chemical properties.

Soil properties	Mean (SE)
pH	6.70(0.20)
Organic matter (mg/g)	6.54(0.81)
Total nitrogen (N, mg/g)	0.20(0.02)
Total phosphorus (P, mg/g)	0.11(0.01)
Total potassium (K, mg/g)	68.54(3.21)
Alkali-hydro nitrogen (mg/kg)	5.87(0.51)
Rapidly-available phosphorus (mg/kg)	3.48(0.03)
Rapidly-available potassium (mg/kg)	56.24(1.20)
