# Supplementary material for: Contrasting effects of prolonged drought and nitrogen addition on growth and non-structural carbohydrate dynamics in coexisting Pinus koraiensis and Fraxinus mandshurica saplings
Source: For Res (Fayettev). 2025 Feb 11;5:e003. doi: 10.48130/forres-0025-0002 (PMC11870304; doi:10.48130/forres-0025-0002)
Supplement: Supplementary file 1 — Supplementary data to this article can be found online. [file forres-0025-0002-S1.zip › 10.48130_forres-0025-0002-Suppl-TableS2.pdf]

**Table S2.** Three-way ANOVA analysis of NSC concentration and its components (soluble sugars and starch) change with different sampling times, drought and nitrogen addition treatments.

| Measurements | Factors | *Fraxinus mandshurica* | | *Pinus koraiensis* | |
|---|---|---|---|---|---|
| | | *F* value | *P*(>F) | *F* value | *P*(>F) |
| Leaf sugar | Time | 9.285 | <0.001*** | 19.802 | <0.001*** |
| | Drought(D) | 0.540 | 0.465 | 2.975 | 0.088 |
| | Nitrogen(N) | 0.293 | 0.747 | 2.405 | 0.097 |
| | D×N | 0.588 | 0.558 | 1.104 | 0.336 |
| Leaf starch | Time | 32.739 | <0.001*** | 121.834 | <0.001*** |
| | Drought(D) | 0.034 | 0.855 | 4.989 | 0.028* |
| | Nitrogen(N) | 3.296 | 0.044* | 8.800 | <0.001*** |
| | D×N | 1.289 | 0.283 | 0.111 | 0.895 |
| Leaf NSC | Time | 25.533 | <0.001*** | 85.542 | <0.001*** |
| | Drought(D) | 0.297 | 0.588 | 0.320 | 0.573 |
| | Nitrogen(N) | 1.423 | 0.249 | 12.022 | <0.001*** |
| | D×N | 1.599 | 0.210 | 0.251 | 0.779 |
| Shoot sugar | Time | 56.425 | <0.001*** | 34.282 | <0.001*** |
| | Drought(D) | 0.340 | 0.562 | 0.167 | 0.684 |
| | Nitrogen(N) | 0.218 | 0.804 | 0.474 | 0.624 |
| | D×N | 0.539 | 0.586 | 0.640 | 0.530 |
| Shoot starch | Time | 62.459 | <0.001*** | 101.056 | <0.001*** |
| | Drought(D) | 3.863 | 0.053 | 0.056 | 0.814 |
| | Nitrogen(N) | 1.817 | 0.169 | 0.450 | 0.639 |
| | D×N | 1.006 | 0.370 | 0.815 | 0.446 |
| Shoot NSC | Time | 13.208 | <0.001*** | 60.321 | <0.001*** |
| | Drought(D) | 1.805 | 0.183 | 0.155 | 0.695 |
| | Nitrogen(N) | 1.865 | 0.162 | 0.711 | 0.494 |
| | D×N | 1.376 | 0.258 | 0.956 | 0.389 |
| Root sugar | Time | 44.945 | <0.001*** | 35.644 | <0.001*** |
| | Drought(D) | 0.004 | 0.951 | 3.153 | 0.080 |
| | Nitrogen(N) | 10.213 | <0.001*** | 1.975 | 0.145 |
| | D×N | 0.441 | 0.645 | 0.584 | 0.560 |
| Root starch | Time | 102.695 | <0.001*** | 59.380 | <0.001*** |
| | Drought(D) | 9.087 | 0.003** | 3.481 | 0.066 |
| | Nitrogen(N) | 10.183 | <0.001*** | 0.771 | 0.466 |
| | D×N | 1.769 | 0.177 | 0.958 | 0.388 |
| Root NSC | Time | 34.502 | <0.001*** | 39.688 | <0.001*** |
| | Drought(D) | 3.795 | 0.055 | 5.755 | 0.019* |
| | Nitrogen(N) | 19.377 | <0.001*** | 0.535 | 0.588 |
| | D×N | 1.264 | 0.288 | 1.356 | 0.264 |
