# Supplementary material for: Contrasting effects of prolonged drought and nitrogen addition on growth and non-structural carbohydrate dynamics in coexisting Pinus koraiensis and Fraxinus mandshurica saplings
Source: For Res (Fayettev). 2025 Feb 11;5:e003. doi: 10.48130/forres-0025-0002 (PMC11870304; doi:10.48130/forres-0025-0002)
Supplement: Supplementary file 1 — Supplementary data to this article can be found online. [file forres-0025-0002-S1.zip › 10.48130_forres-0025-0002-Suppl-TableS3.pdf]

Table S3. Two-way ANOVA analysis of nitrogen (N) content change with drought and nitrogen addition treatments at specific sampling times. *F* value of ANOVA is given, the followed ***, ** and * indicates significant difference between different treatment levels at *P*<0.001, *P*<0.01 and *P*<0.05, respectively (*F* value in bold if *P*<0.05). *F.mand*: *Fraxinus mandshurica*, *P.kora*: *Pinus koraiensis*.

Species	Times	Factors	Leaf N	Shoot N	Root N	Weighted N
F.mand	S1	Drought(D)	24.090***	4.743	0.399	0.082
		Nitrogen(N)	53.210***	0.230**	4.558*	1.598
		D×N	14.630***	1.483	3.102	5.492*
	S2	Drought(D)	4.655	0.760	0.806	0.770
		Nitrogen(N)	47.654***	4.931*	13.252***	15.823***
		D×N	1.254	0.641	4.173*	3.282
	S3	Drought(D)	No data	5.379*	5.162*	6.118*
		Nitrogen(N)		4.025*	5.978*	8.875**
		D×N		0.382	2.771	3.073
	S4	Drought(D)	11.088**	29.331***	0.931	2.382
		Nitrogen(N)	7.975**	5.737*	24.122***	26.304***
		D×N	2.220	20.098***	3.792	5.155*
	S5	Drought(D)	3.180	0.713	1.961	3.540
		Nitrogen(N)	42.796***	22.499***	46.666***	51.050***
		D×N	0.383	2.733	0.989	0.556
P.kora	S1	Drought(D)	0.080	0.550	6.190*	7.654*
		Nitrogen(N)	0.501	2.282	3.212	10.602**
		D×N	6.429*	0.737	10.595**	2.014
	S2	Drought(D)	1.815	0.005	0.069	0.061
		Nitrogen(N)	4.228*	4.020*	2.029	2.988
		D×N	0.155	0.433	0.644	0.549
	S3	Drought(D)	4.455	0.649	0.521	6.115*
		Nitrogen(N)	17.102***	5.871*	5.720*	24.664***
		D×N	1.367	1.669	4.001*	3.713
	S4	Drought(D)	1.007	1.456	0.003	0.088
		Nitrogen(N)	24.410***	3.353	15.920***	14.062***
		D×N	0.458	0.352	1.137	0.035
	S5	Drought(D)	3.009	5.669*	0.110	4.251
		Nitrogen(N)	10.076**	7.972**	4.432*	9.449**
		D×N	0.580	7.492**	2.449	0.821
