# Supplementary material for: Contrasting effects of prolonged drought and nitrogen addition on growth and non-structural carbohydrate dynamics in coexisting Pinus koraiensis and Fraxinus mandshurica saplings
Source: For Res (Fayettev). 2025 Feb 11;5:e003. doi: 10.48130/forres-0025-0002 (PMC11870304; doi:10.48130/forres-0025-0002)
Supplement: Supplementary file 1 — Supplementary data to this article can be found online. [file forres-0025-0002-S1.zip › 10.48130_forres-0025-0002-Suppl-TableS4.pdf]

Table S4. Nitrogen content (N, mg/g) in shoot and root (Mean, SD) of two species. The followed upper-case letters represent significant differences in means among drought treatments, while lower-case letters represent significant differences between nitrogen treatments (N), or between all six combinations in case of a D×N interaction exists, tested with Tukey post-hoc test. The different sampling times: S1: August, 2017; S2: September, 2017; S3: January, 2018; S4: June, 2018; and S5: August, 2018.

Treatment	Times	*Fraxinus mandshurica*		*Pinus koraiensis*	
		Shoot N	Root N	Shoot N	Root N
N0.CK	S1	0.065 (0.013)Ab	0.091 (0.045)Ab	0.345 (0.023)	0.434 (0.039)b
N1.CK		0.134 (0.054)Ab	0.375 (0.213)Aab	0.375 (0.02)	0.431 (0.108)b
N2.CK		0.284 (0.14)Aa	0.708 (0.249)Aa	0.532 (0.178)	0.344 (0.127)b
N0.Drought		0.073 (0.042)Ab	0.341 (0.105)Ab	0.453 (0.131)	0.361 (0.032)b
N1.Drought		0.022 (0.007)Ab	0.259 (0.039)Aab	0.418 (0.092)	0.45 (0.083)b
N2.Drought		0.168 (0.078)Aa	0.397 (0.337)Aa	0.491 (0.098)	0.69 (0.062)a
N0.CK	S2	0.185 (0.092)Ab	0.505 (0.145)b	0.269 (0.016)Ab	0.656 (0.021)
N1.CK		0.393 (0.086)Aab	0.988 (0.407)ab	0.303 (0.052)Aab	0.665 (0.111)
N2.CK		0.358 (0.065)Aa	1.032 (0.468)ab	0.484 (0.159)Aa	0.775 (0.146)
N0.Drought		0.11 (0.032)Ab	0.198 (0.054)b	0.261 (0.066)Ab	0.574 (0.219)
N1.Drought		0.263 (0.167)Aab	0.462 (0.188)b	0.363 (0.121)Aab	0.778 (0.148)
N2.Drought		0.398 (0.241)Aa	1.478 (0.341)a	0.421 (0.184)Aa	0.799 (0.173)
N0.CK	S3	0.211 (0.155)Ab	0.406 (0.163)Ab	0.276 (0.022)Ab	0.395 (0.136)b
N1.CK		0.31 (0.162)Aab	0.943 (0.473)Aab	0.309 (0.073)Ab	0.577 (0.044)ab
N2.CK		0.482 (0.08)Aa	1.682 (0.359)Aa	0.339 (0.065)Aa	0.587 (0.125)ab
N0.Drought		0.134 (0.064)Bb	0.467 (0.137)Bb	0.297 (0.053)Ab	0.532 (0.078)ab
N1.Drought		0.173 (0.084)Bab	0.621 (0.325)Bab	0.275 (0.012)Ab	0.439 (0.04)ab
N2.Drought		0.276 (0.176)Ba	0.715 (0.61)Ba	0.408 (0.039)Aa	0.681 (0.075)a
N0.CK	S4	0.117 (0.013)bc	0.739 (0.186)Ab	0.308 (0.014)	0.177 (0.054)Ab
N1.CK		0.073 (0.02)bc	0.651 (0.014)Ab	0.315 (0.035)	0.241 (0.019)Ab
N2.CK		0.054 (0.014)c	0.988 (0.171)Aa	0.349 (0.031)	0.331 (0.071)Aa
N0.Drought		0.082 (0.049)bc	0.584 (0.028)Ab	0.315 (0.072)	0.202 (0.064)Ab
N1.Drought		0.155 (0.039)b	0.762 (0.074)Ab	0.334 (0.027)	0.189 (0.052)Ab
N2.Drought		0.276 (0.053)a	1.193 (0.112)Aa	0.397 (0.055)	0.353 (0.007)Aa
N0.CK	S5	0.232 (0.013)Ab	0.731 (0.012)Ac	0.343 (0.037)b	0.394 (0.097)Ab
N1.CK		0.355 (0.051)Aa	0.933 (0.052)Ab	0.361 (0.069)b	0.564 (0.126)Aab
N2.CK		0.359 (0.04)Aa	1.148 (0.046)Aa	0.356 (0.053)b	0.626 (0.025)Aa
N0.Drought		0.276 (0.027)Ab	0.775 (0.07)Ac	0.338 (0.031)b	0.522 (0.059)Ab
N1.Drought		0.318 (0.029)Aa	0.926 (0.017)Ab	0.343 (0.04)b	0.541 (0.09)Aab
N2.Drought		0.39 (0.014)Aa	1.275 (0.177)Aa	0.619 (0.136)a	0.559 (0.034)Aa
