# Supplementary material for: Contrasting effects of prolonged drought and nitrogen addition on growth and non-structural carbohydrate dynamics in coexisting Pinus koraiensis and Fraxinus mandshurica saplings
Source: For Res (Fayettev). 2025 Feb 11;5:e003. doi: 10.48130/forres-0025-0002 (PMC11870304; doi:10.48130/forres-0025-0002)
Supplement: Supplementary file 1 — Supplementary data to this article can be found online. [file forres-0025-0002-S1.zip › 10.48130_forres-0025-0002-Suppl-FigureS1.pdf]

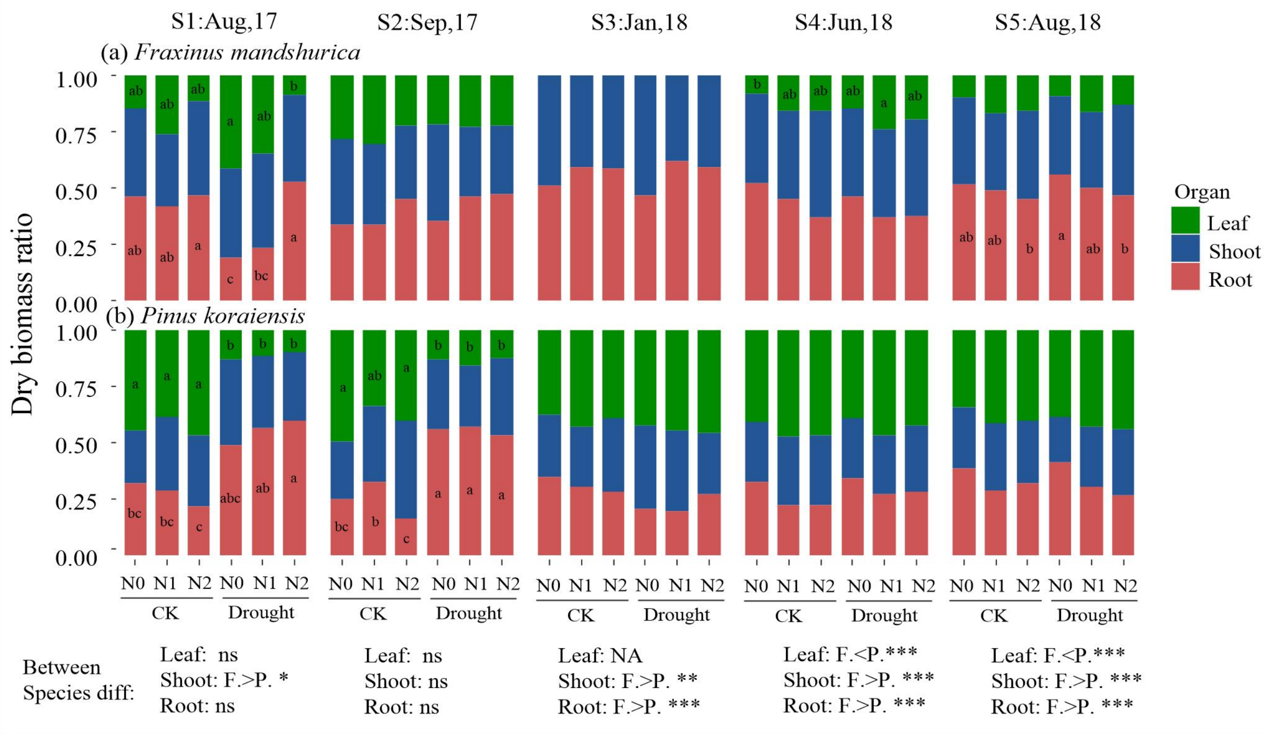

Fig. S1 The relative biomass allocation between leaf, shoot and root in *Fraxinus mandshurica* and *Pinus koraiensis*. The lower-case letters on the bars represent significant differences within each organ, between all six combinations, tested with Tukey post-hoc test. Difference between species were showed below each sampling time. > or < means significant difference, ns showed no significant, for leaf at S3 (Jan, 2018), NA for no comparable leaf data for test.
